# Ten years of global disease detection and counting: program accomplishments and lessons learned in building global health security

**DOI:** 10.1186/s12889-019-6769-2

**Published:** 2019-05-10

**Authors:** Joel M. Montgomery, Abbey Woolverton, Sarah Hedges, Dana Pitts, Jessica Alexander, Kashef Ijaz, Fred Angulo, Scott Dowell, Rebecca Katz, Olga Henao

**Affiliations:** 1 Division of Global Health Protection, Center for Global Health, Centers for Disease Control and Prevention, 1600 Clifton Road N.E., Mailstop H16-+5, Building 16, Atlanta, GA 30329 USA; 2 Present Address: National Center for Emerging and Zoonotic Infectious Diseases, Office of the Director, Centers for Disease Control and Prevention, Atlanta, GA USA; 3 Present Address: Pfizer Vaccines, Portland, OR USA; 4 Center for Global Health Science and Security, Georgetown University, Washington, DC USA; 5 Office of Public Health Science and Surveillance, Centers for Disease Control and Prevention, Atlanta, GA USA; 6 National Center for Health Statistics, Division of Vital Statistics, Centers for Disease Control and Prevention, Atlanta, GA USA; 7 Bill and Melinda Gates Foundation, Seattle, WA USA

## Introduction

Worldwide, infectious diseases continue to emerge at an alarming pace, due to numerous factors including microbial adaptation, increasing human population migration, urbanization, conflict and instability, intensified animal-human interface, and habitat perturbation [1–6]. The litmus test for an effective national public health program is its ability to be ready to initiate an effective response for an unknown emerging or re-emerging infectious disease or public health event. The most impactful global health programs are built with the understanding that they must be able to help countries strengthen core public health capacity so that new threats can be detected and contained before they become international crises that increase morbidity and mortality, adversely impact the health and livelihoods of individuals and populations, disrupt travel, interfere with global trade and economies, or even lead to political destabilization [6, 7].

This is the basis for all global health security work and has been the mission of CDC’s Global Disease Detection (GDD) program since its inception in 2004. As one of the first steps through which CDC systematically approached global health security, the GDD program was designed to bring resources together to promote a broader approach to preparing countries for any infectious disease threat that could occur [8]. Today, after more than a decade of partnerships in groundbreaking science, disease detection, and response to the world’s most urgent public health threats, lessons from the GDD program as a precursor to global health security offer the global health community one model for collective success. This supplement is dedicated to highlighting a sample of successes achieved and lessons learned through the GDD program throughout its 10+ years of implementation.

## Beginnings and principles

The idea for the GDD program took shape against the backdrop of the 2002–2003 severe acute respiratory syndrome (SARS) epidemic, which affected more than 8000 people in 29 countries and cost the world more than $40 billion US dollars [9, 10]. In 2004, the U.S. Congress authorized funding for CDC to establish the GDD program [8]. Using existing research infrastructure developed as part of CDC’s International Emerging Infectious Diseases Program, the GDD program was developed to “promote global health security by building capacity to rapidly detect and contain emerging health threats [8, 11, 12]. Since its inception, the GDD program has held a broader more cross -cutting mandate than previous CDC programs. Rather than focusing on a single disease or issue, the GDD program helps prepare countries for any emerging or reemerging infectious disease outbreak or significant public health event.

**Fig. 1:**
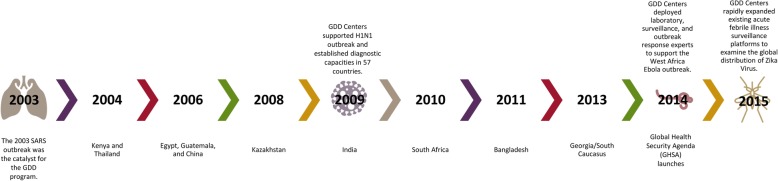
Timeline of the Evolution of the Global Disease Detection Program

To fulfill its mission, the GDD program uniquely established a network of Regional Centers (GDD RCs) to help countries rapidly and effectively address public health threats. These international centers formed a worldwide base of health security through scientific evidence-based capacity building and creating strong, trusted ties with partner countries (Fig. 1).

The mandates of the GDD RCs were to help develop a strong workforce of epidemiologists and laboratorians; enhance or promote the One Health concept [13] by encouraging multi-sectoral collaborations between Ministries of Health and Ministries of Agriculture; and build and expand state-of-the-art laboratory capacity for detection of newly emerging infectious diseases in addition to strengthening basic laboratory diagnostic capabilities. To date, GDD RCs have provided expert consultations, supported outbreak response, and offered epidemiology and laboratory training in more than 103 countries.

**Fig. 2:**
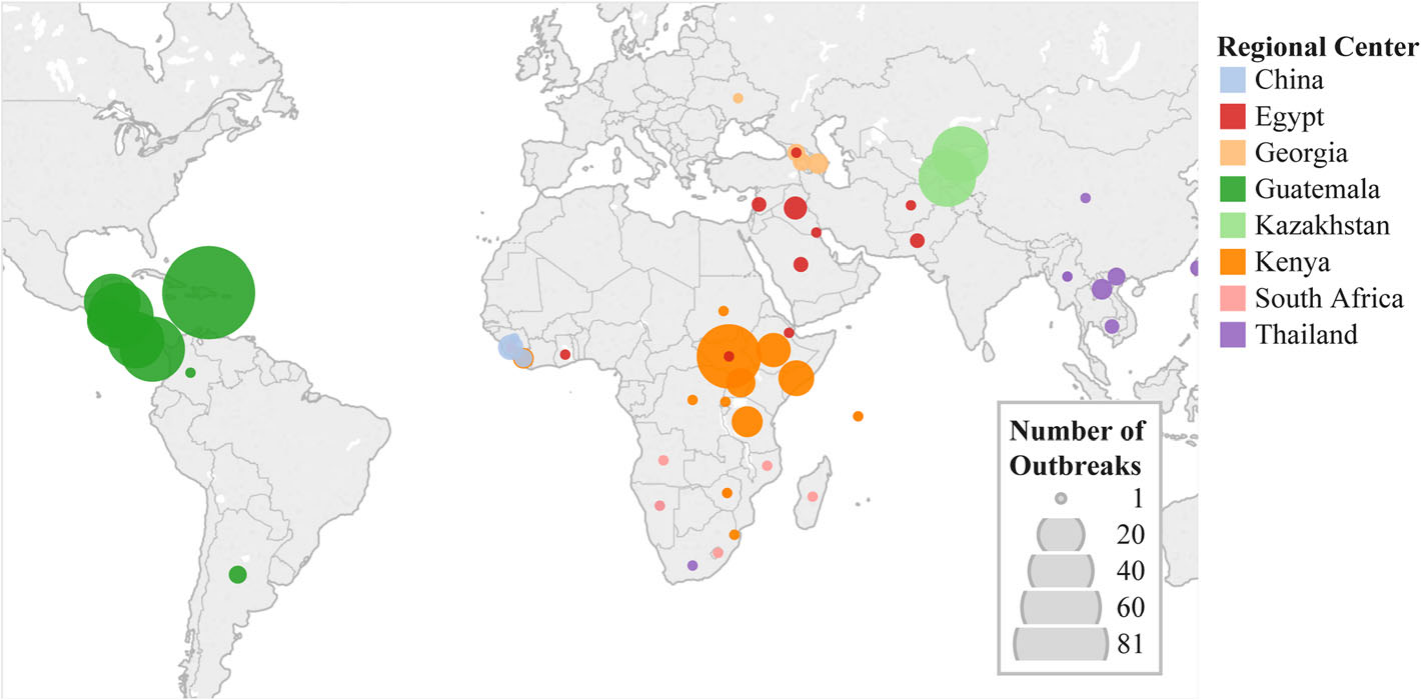
Map of GDD Regional Centers (GDD RCs) and outbreak support provided by the GDD RCs from 2007 to 2016. Color corresponds to the GDD RC that provided support, while size corresponds to the number of outbreaks supported in each country. Note: Outbreaks responded to in the home country of each GDD RC were not included in this map

Ten GDD RCs existed (Fig. 2) as of January 2018, representing the Americas, Africa, and Asia – including the Indian subcontinent and Southeast Asia. Selection of countries for placement of GDD RCs was based on a number of factors, including: 1) country interest in hosting a GDD RC, including track-record of previous successful collaborations with US government agencies 2) high burden or perceived high burden of infectious diseases in the country or region, 3) potential for infectious disease emergence, and 4) a need to strengthen or improve public health infrastructure to detect and respond to infectious disease outbreaks.

An early insight was that the baseline public health infrastructure varied from country to country. At a minimum, all were in need of workforce development (i.e. trained field epidemiologists, public health laboratorians, data analysts and health communicators), improvements in the ability to develop complex laboratory diagnostics, and creation or improvement of disease surveillance, including specimen transport systems and integration of laboratory and surveillance data into an adequate response system [12]. To meet this variety of needs, CDC placed experienced medical epidemiologists, laboratorians, veterinarians, and public health specialists in a number of the GDD RCs [12].

The work of the GDD RCs has been guided by two overarching objectives or principles: 1) to conduct cutting edge public health science, including original research, and to generate solid data to inform public health policy decisions, and help guide public health capacity building, and 2) to have forward-deployed assets or pre-positioned staff, equipment and supplies to rapidly support the host country government’s ability to respond to outbreaks and prevent further spread of disease within and outside the borders of the country.

### GDD and Global Health security

Global health preparedness is a priority worldwide, as evidenced by the adoption of the International Health Regulations (IHR) in 2005, and the subsequent work of over 60 nations, the U.S. Government, CDC, the World Health Organization (WHO), to advance the Global Health Security Agenda (GHSA) [14]. GHSA, launched in February 2014, is a commitment between countries to marshal resources, expertise, and technical assistance to build core public health infrastructure around the world and monitor progress using specific metrics and targets.

The ultimate goal of the GHSA is to better prepare for epidemics and pandemics and to help countries meet their commitments to the WHO IHR, 2005 [15] and the World Organization of Animal Health’s (OIE) Performance of Veterinary Services Pathway [16]. The GDD program’s value in helping countries achieve IHR goals was solidified in December 2009, when the program – although a relatively small program with a modest budget – was designated by WHO as a Collaborating Center for Implementation of IHR National Surveillance and Response Capacity [17].

The GDD program contributes to global health security efforts in much the same way as GHSA by strengthening the world’s core public health capacity, ultimately helping countries achieve IHR compliance. The program serves the countries in which it resides, as well as neighboring countries, with the expertise and support needed to prevent, detect, and respond to any public health threat.

## A look at the data: GDD program activities and accomplishments

The GDD program has collected data for both quantitative and qualitative indicators to monitor and evaluate the progress and effectiveness of its regional centers since 2006, with some additional indicators added in 2007 and 2017. These indicators cover multiple topic areas including consultations, outbreak investigations, trainings and workforce development, pathogen discovery, new diagnostic testing capacity, surveillance, networking, and publications.

Data associated with each GDD RC capture both efforts and outcomes in country and support to other nations. Additional CDC datasets with information on human assets deployed during the Ebola epidemic and data sharing during the Zika epidemic were also included in these analyses. The three datasets (GDD program indicators [2006–2016], Ebola, and Zika) were analyzed using Tableau Software, version 10.3.2. Findings were validated with targeted outreach to CDC personnel.

### Outbreak response

From 2006 to 2016, GDD RCs responded to 2051 outbreaks around the world. Each outbreak response corresponded to a specific event regardless of the number of cases identified during the outbreak – for example, a single case of rabies and an outbreak of dengue resulting in 8000 cases were both considered single outbreak events. Outbreaks also included events in animals, such as H5N1 in poultry, West Nile Virus in horses, and rabies in dogs, as well as responses related to the environment such as pesticide poisonings and natural disasters. One quarter (509 of 2051) of all outbreaks that GDD RCs responded to between 2006 and 2016 occurred outside of the GDD RCs’ countries of origin (Fig. 2).

**Fig. 3:**
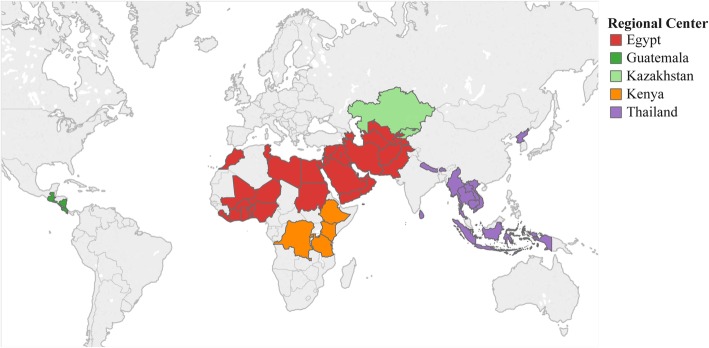
Map of 57 countries that gained laboratory testing capacity to detect H1N1 during the 2009 H1N1 pandemic, in partnership with Regional Centers. Color corresponds to the RC that supported H1N1 laboratory capacity building. Note: Map does not include countries that had existing capacity to detect H1N1. RCs in India (2009), South Africa (2010), Bangladesh (2011), and Georgia (2013) were established during or after 2009

Among one of the most important contributions of the GDD RCs have been responses during the recent WHO-declared Public Health Events of International Concern (PHEICs). Shortly after the April 2009 declaration of H1N1 as a PHEIC, GDD RCs in Egypt, Guatemala, Kazakhstan, Kenya, and Thailand partnered with 57 countries to improve and/or establish diagnostic laboratory capacity to detect H1N1 (Fig. 3); 46 of these (80%) partnerships or interactions occurred from May 1–June 1, 2009.

**Fig. 4:**
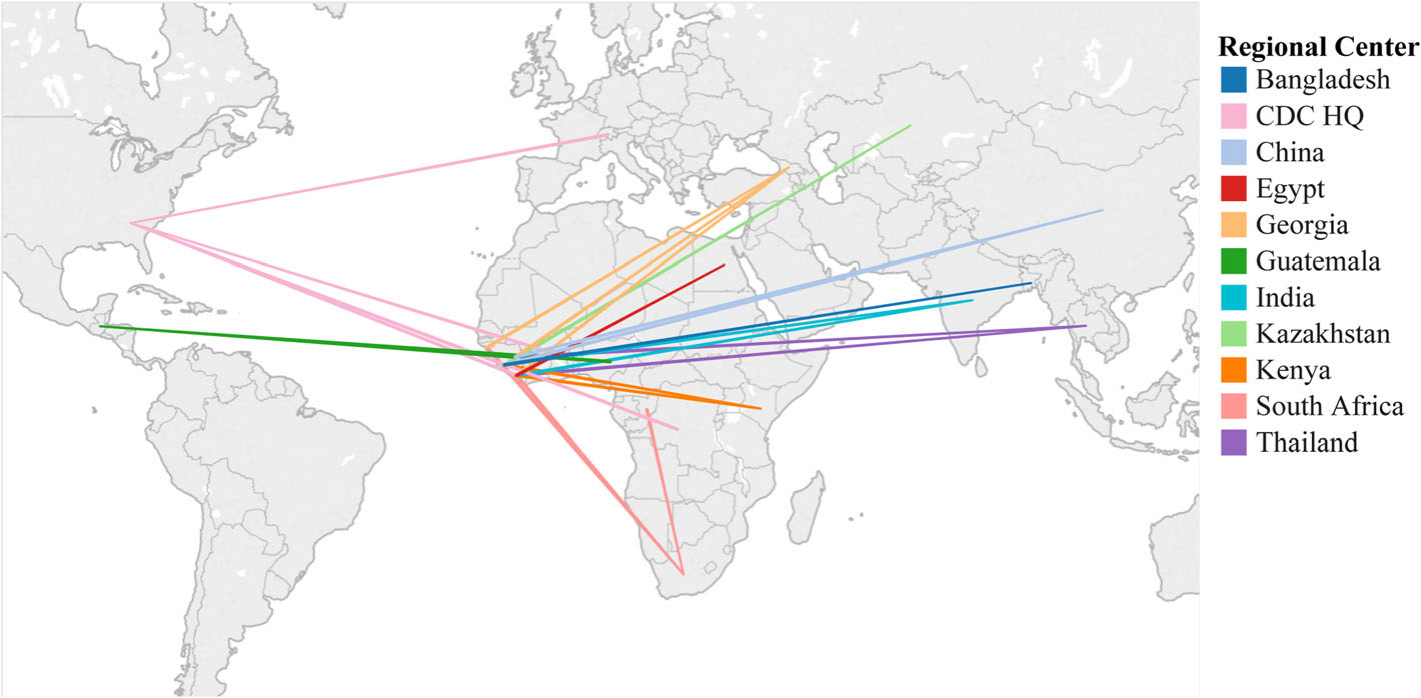
Map showing the deployment of 45 Regional Center staff to countries in West Africa and Geneva, Switzerland from CDC’s 10 Regional Centers around the world and from CDC’s headquarters in Atlanta, GA in response to the 2014 Ebola epidemic. Note: Map does not include multiple deployments

During the 2014–2015 Ebola outbreak in West Africa, all 10 GDD RCs, as well as CDC Headquarters in Atlanta, deployed GDD program personnel to aid in the response effort (Fig. 4). In total, 45 individuals associated and stationed within the GDD RCs were among the first responders to be deployed; 37 of these individuals deployed to Liberia, Guinea, and Sierra Leone, while others deployed to the Republic of Congo, Guinea-Bissau, DRC, Nigeria, Benin, and Switzerland. In addition, 13 of the 45 (29%) individuals deployed were host country nationals or locally employed staff.

GDD RCs not only directly supported Ebola response efforts but also prepared their respective and neighboring countries for possible importation and spread of the virus within their borders. Using an assessment tool developed by CDC, GDD Guatemala conducted an Ebola preparedness assessment for 12 Latin American nations, while GDD Egypt trained 13 participants from Jordan, Morocco, Lebanon, Tunisia, and Egypt on Ebola preparedness. GDD Bangladesh collaborated with a large number of non-governmental private sector groups to develop standard operating procedures for Ebola case management and response; similarly, GDD Thailand collaborated with health ministers from 13 countries to develop a strategic framework for enhancing partnership on Ebola preparedness and response. Laboratory testing capacity for Ebola was increased by GDD India, and airport screening protocols and border security were improved by GDD Kenya. Meanwhile, GDD RCs in South Africa and China focused on increasing communication platforms for the dissemination of information concerning Ebola. Furthermore, the Chinese Field Epidemiology Training Program (FETP) deployed 23 current and former trainees for the first time and U.S. CDC locally engaged country staff were deployed to Sierra Leone to help transfer laboratory technology to the Chinese laboratory in-country.

The GDD RCs were well poised to act during the 2014–2017 Zika epidemic. Using a combination of funding sources, including Zika supplemental funding provided through partnership with the US Agency for International Development and GDD core funding, eight of the 10 GDD RCs (Guatemala, Kenya, Thailand, South Africa, India, Bangladesh, China, and Egypt) were able to use their existing acute febrile illness surveillance systems to implement a global, network-wide surveillance activity to examine the global distribution of Zika virus. The timely introduction of Zika testing into existing GDD supported surveillance platforms allowed for the rapid identification and characterization of some of the first Zika cases in Guatemala and India [18]. The Guatemala, Kenya, and Thailand RCs were also able to quickly design and implement studies to examine the effect of Zika virus infection in pregnant women and their babies, and the Guatemala RC was able to initiate activities to examine potential long-term outcomes of infection. Leveraging existing platforms allowed for faster implementation of activities. It also generated important lessons for future responses, such as the need to consider how differences among protocols can affect comparability of results across countries, and highlighted the potential benefits of centralized coordination of surveillance and research.

### Laboratory capacity

One of the strengths of the GDD RCs has been their ability to increase laboratory capacity for identification of threats, including identifying new pathogens to the world or pathogens new to a region.

Of the 2051 outbreak responses, the GDD RCs provided laboratory support in 1363 (66%). Of these 1363 laboratory-supported outbreak responses, 1153 (85%) resulted in a confirmed etiology or cause of the outbreak. In the same 10-year period, 381 pathogen-specific tests were newly established or updated by GDD RCs in 62 countries through a program of deliberate technology transfer. Examples included tests for pathogens of international concern or pandemic potential (e.g. H1N1, H5N1, H7N9, MERS coronavirus, chikungunya, and Ebola), respiratory pathogens (e.g. adenovirus, rhinovirus, coronavirus, and RSV), acute febrile illness pathogens (e.g. Q Fever/*Coxiella burnetii*, *Leptospirosis*, *Brucella*, *Rickettsia*, and West Nile Virus), food and waterborne pathogens (e.g. *Escherichia coli*, *Salmonella*, *Shigella*, *Listeria*, and *Campylobacter*), and others (e.g. *Bartonella* species, Botulinum neurotoxins, arboviruses, arenaviruses).

In collaboration with local and international partners, GDD RCs conducted groundbreaking work on 79 organisms during 2007–2016, including detecting 62 organisms new to their respective regions, discovering 11 organisms and pathogens new to the world, and identifying 5 pathogens with a new mode of transmission (Table 1). Examples of pathogens new to their respective regions included novel influenza viruses (H1N1, H5N1, H3N2, H9N2), arboviruses (dengue serotype 3, Chikungunya virus, Zika virus, West Nile virus, Tahyna virus, Babanki virus, Ndumu virus, Sindbis-like virus, Usutu virus, Sandfly fever Sicilian virus) [19, 20], and bacterial and parasitic pathogens (Q fever/*Coxiella burnetii*, *Leishmania* species, and *Legionella longbeacheae*) [21].

**Table 1:** Table of newly discovered organisms by GDD Regional Centers from 2006 to 2016, categorized by nature of detection

Total New Pathogens Detected/Discovered	79
New to the World (Organism)	5
New to the World (Pathogen)	6
New Mode of Transmission	6
New to the Region	62

### Surveillance, epidemiology, and data capacity

In 2017, the GDD program began collecting data on the number and type of surveillance platforms and the number of people enrolled or captured in active or passive disease surveillance systems. GDD RCs’ activities currently cover more than 109,000,000 people through various types of surveillance platforms. GDD RCs have established more than 288 unique surveillance sites monitoring disease syndromes and specific illnesses such as acute febrile illness, respiratory disease, Japanese Encephalitis, and Nipah Virus. The syndrome most commonly responded to by GDD RCs from 2007 to 2016 was gastrointestinal illness (diarrhea, vomiting), followed by influenza-like illness (ILI) and acute/undifferentiated febrile illness (A/UFI). Increased ILI cases in 2009 were due to the pandemic of H1N1 and outbreaks of H5N1, while increased gastrointestinal illnesses in 2013 corresponded to cases of cholera in Kenya, and increased A/UFI in 2014 correlated with the dengue and chikungunya outbreaks in East Africa.

The types of disease surveillance platforms implemented via the GDD RCs include event-based, sentinel, facility-based, and population-based surveillance. The ability to conduct population-based surveillance is particularly important because it often provides the most accurate information on the burden of infectious disease syndromes, as it allows for the calculation of their incidence, which is the number of cases among a known population size during a standard period of time. GDD RCs in China, Egypt, Guatemala, India, Kenya and Thailand have conducted population-based surveillance over the course of the 10-year period [22–27]. The incidence rates generated via these platforms are important measures of disease burden because they can be compared across different locales. Examples of uses of data derived from population-based surveillance include the comparison of rates of disease in rural areas with rates in urban areas and the monitoring of impact of interventions or control strategies.

### Workforce development

The GDD program recognizes that a strong workforce lies at the core of effective emergency response. From 2006 to 2016, the GDD RCs trained more than 115,000 multi-disciplinary public health professionals through 3399 unique training sessions. The subject matter experts from across CDC headquarters, as well as highly trained medical epidemiologists, laboratorians, veterinarians, and public health specialists stationed within the GDD RCs lead formal training programs, such as the Field Epidemiology Training Program (FETP), offer informal on-the-job-training and provide mentorship to local counterparts [28]. In addition to leading training opportunities such as tabletop exercises and data analysis workshops, GDD RCs capitalize on CDC subject matter expertise around the agency to provide disease-specific guidance and training. This development of workforce capacity at the local level is integral to identifying and containing public health threats at their source.

The graduates of FETPs in GDD RCs from 2006 to 2016 responded to many of the outbreaks recorded, leading to proper identification of the source for many of the outbreaks, detection of additional cases/determining the full scope of the outbreaks, and classification or discovery of existing or novel risk factors of disease transmission. More often than not, these graduates continue to practice public health in-country after graduating [28].

### Research and support

GDD RCs served as the platform for subject matter experts and researchers across CDC and through their effort provided a total of 4253 public health consultations from 2006 to 2016. Consultations varied widely in scope, type of collaborators, and length of partnership. For example, GDD Kenya regularly provided consultations on health issues affecting refugees in Kenya, Ethiopia, Uganda, and Tanzania, while GDD India teamed up with the National Institute of Mental Health and Allied Sciences (NIMHANS) to work on an acute encephalitis syndrome network. Regional Centers such as GDD Egypt, GDD Kazakhstan, and GDD Georgia conducted laboratory assessments at laboratories and hospitals, recommended laboratory equipment for national blood banks, and advised on infection control procedures, respectively, with subject matter expertise support from CDC headquarters.

Furthermore, the GDD RCs supported the use of technology by collaborating with provincial satellite TV channels to communicate risks such as hand, foot, and mouth disease (HFMD) and H1N1 in Vietnam – as was the case with GDD China – and by providing technical support for an electronic surveillance platform in Panama, coordinated out of the GDD RC in Guatemala. Finally, GDD RCs worked with a number of collaborators (i.e., GDD South Africa with National Park staff, GDD Bangladesh with live-bird market workers, and GDD Thailand with veterinarians) on a variety of One Health projects.

The GDD program collaborates with experts across CDC, maximizing the subject matter expertise residing in the agency, and with ministries of health and international partners. From 2006 to 2016, GDD RC staff authored or co-authored a total of 877 peer-reviewed articles and 1416 other significant documents, such as policy documents, position papers, and training manuals. These publications address disease-specific outbreaks and emergencies, surveillance and laboratory science, and cross-cutting priorities related to disease threats. These publications show the diversity and strong scientific foundations of GDD’s work.

## Discussion and lessons learned

The original overarching goal and purpose of the GDD program was to improve global capacity within partner nations to prevent emerging infectious disease threats at the site of origin, rapidly detect disease events, and respond to outbreaks to mitigate the consequences to the population. The accomplishments of the GDD RCs highlight examples of many firsts: diseases detected before they became significant threats; additions of new laboratory tests to identify the cause of illness; vital workforce training programs begun and expanded; as well as faster, smarter response to outbreaks because of the capacity the program helped build in-country.

As previously noted, the GDD program offered an early strategic approach to global health security efforts as countries worked to meet their obligations under the IHR [15]. When the IHR [15] were adopted in 2005, the GDD program was uniquely positioned to help close the critical gap between global public heath capacities defined in the IHR [15] and the ability of many member states to meet these requirements. Over a decade after implementation of the IHR [15] more than 60 countries have extended their commitment to strengthening global public health capacity through the GHSA [14]. The GDD program again offered a framework for – and experience in – implementing the cross-cutting public health systems needed to meet the targets set forth by both IHR and GHSA.

The GDD program exemplifies the work that CDC has done to improve global health outcomes and enhance global health security specifically as part of the core functions of the organization. The GDD program unites the resources of the United States and its international partners to provide technical assistance, logistical support, and funding through regional networks and intergovernmental organizations. Through this work, we have increased the capacity of the global public health workforce to identify and contain threats.

It is critical to note that the value of the in-country work done by the GDD program extends beyond stopping outbreaks. Partnerships and relationships formed through the program have contributed to health diplomacy abroad. These critical ties extend our ability to respond in times of crisis, and play an additional role in strengthening other initiatives and programs that protect public health. Public health programs like GDD have served as inroads to connection in fragile areas, such as those facing political instability and conflict, because they remove barriers to collaboration by addressing universally acknowledged health needs.

### One tested model for the future of global health security

The GDD program’s efforts over the last decade to improve global public health capacity have, indeed, moved us forward. Measurable progress has been made within a focused, but limited, scope. For progress to continue, however, CDC and the global health community must go beyond our initial efforts and work more broadly to confront challenges and embrace opportunities that arise. The GDD program has given us the following important lessons that can inform our next steps:

1. *Create multiregional connectivity.* Strong networks can harness a variety of strengths, share resources, and connect across disciplines toward common goals. A major success of the GDD program has been to create regional platforms where subject matter experts can engage with one another and programs can break free of their silos. Moving from siloed to shared approaches also enhances collaboration on science and research, thereby strengthening the foundation for public health action. Global networks have been created by GDD, and more recently with GHSA, in recognition that shared risk means shared responsibility, and the best way to achieve success is by working together to ensure our collective health, safety, and security.
2. *Adopt consistent goals and measures*. From the beginning, the GDD program has applied a consistent set of goals and metrics to track progress over time and across programs. The world’s global health security efforts are also seeing the benefits of instituting consistent targets, as well as frameworks for measuring success against those targets. Over the past few years, the WHO Joint External Evaluations have become a valuable tool to track progress on global health security initiatives, both past-to-present and country-to-country [29]. Evaluation is a key part of recognizing accomplishments and is critical to finding gaps we must still address. Only once we know where we stand can we take action to implement successful programs and point them in the right direction to reduce our identified vulnerabilities.
3. *Deploy the power of science and data*. Cutting-edge scientific research has always been at the core of the GDD program’s mission. Scientific data are the tool we use to detect, respond, and to halt or prevent outbreaks and to inform policy changes that protect public health globally. Scientific research helps partners make evidence-based decisions and implement effective local solutions that eliminate outbreaks at their source. Additionally, taking an active role in teaching others how to capture, analyze, and effectively use public health data creates a workforce capable of rapidly recognizing and responding to threats. Future scientific progress will require not only improved connection across scientific disciplines, but also sustained and dedicated commitment to a unified scientific strategy.
4. *Build trusted partnerships.* The GDD program’s success has relied on strong partnerships. The program’s longstanding presence in regions across the globe has proven that in-country engagement leads to trust. This trust becomes particularly valuable in outbreak response, as global partners rely on CDC data and expertise as a resource that saves lives. Strong partnerships at all levels are critical to global health security, and the process of creating GDD RCs has formed and strengthened partnerships at all levels – government-to-government relationships, collaboration with other organizations and non-governmental organizations (NGOs), and local and personal connections – that can be leveraged to address critical public health priorities.
5. *Build for flexibility*. Cross-cutting public health programs give us the ability to respond to any crisis, regardless of cause. Strong core systems and connected resources can pivot when needed to address emerging or reemerging threats. As threats change, and as science changes, funding tied to a single disease may prove limiting in its scope. Conversely, investment in core public health capacity ensures that a single mission does not dictate the longevity or capacity of a program, and that we can continue to maintain and grow our valuable resources, expertise, and connections. Flexible, nimble systems are our best answer to an unpredictable future.

### Ongoing challenges

While there have been many successes and substantial impacts made by GDD RCs, there have also been significant challenges recognized. Some of these have impacted the ability of the GDD program to accomplish one of its primary goals: helping countries achieve IHR compliance.

Despite the global prominence of infectious diseases, there are few rigorous and precise estimates of the burden and etiology of key infectious disease syndromes in developing countries [30, 31]. Some of the problems in measuring the burden of these diseases in developing countries have included poor access to the clinical facilities, lack of accurate or available laboratory diagnostics, and absence of population-based surveillance systems needed to accurately assess incidence rates. Accurate information on burden of the most important infectious disease syndromes is needed by ministries of health and public health policy decision-makers to set current priorities for optimal use of limited resources for public health programs. Efforts to assist our partner countries in building national laboratory and surveillance systems have been significantly hampered by insufficient resource allocation – both financial and staff time. This has led to a greater recognition of the actual time and money required to develop and maintain such systems.

Another challenge has been the need for better coordination and communication of a unified mission and objective that is supported, fully adopted and implemented in all of the GDD RCs. In some instances, lack of clarity on adopting and implementing a unified mission led to a divergence of operations and a mixture of activities driven, in many cases, by individual investigator interests and expertise in country. The inability to have every kind of public health expertise represented among country-based staff highlights the need for sustained and active scientific engagement across the agency. The public health science conducted through such activities has been commendable; however, the data generated has not always been completely successful in informing policy for ministries of health (i.e., vaccine coverage, educational campaigns targeting high-risk populations, improvement or development of vector-control programs). More needs to be done to ensure that data are applied to their full potential in improving the health of the populations served.

Finally, although there have been some cross GDD RC projects (e.g., Use of a multipathogen Taqman Array Card to identify the etiology of community-acquired pneumonia, C. Van Beneden *pers com*), overall, it has been a challenge for the GDD RCs to link across a network of regional offices or platforms to implement unified protocols or projects (i.e. estimating burden of a specific disease, measuring the effect of a specific medical countermeasure, etc.) in multiple countries, throughout multiple populations, in diverse ecologies, and among unique cultural settings. Building the capacity to do this could strengthen the overall goals of global health security to prevent, detect and respond to health threats.

## Conclusion

The GDD program is part of a long and significant history at CDC of protecting health globally, ranging from smallpox eradication, polio elimination, HIV, malaria, and cholera control to emergencies including SARS, H1N1, Ebola, and Zika. As this history shows us, global health is never static, and the work is not finished.

As we look to the future, our biggest challenge remains the unknown. Health threats will continue to take us by surprise. The nature of disease means that we cannot always predict what the next outbreak will be, or where and how it will spread. Ever-increasing interconnection across the globe means that when the next outbreak does take hold, it will be capable of spreading rapidly. To stop it, we will need systems in place that are sensitive enough to signal a new health threat, specific enough to pinpoint problems and focus resources, and flexible and connected enough to protect the world’s economic and social wellbeing. We must recognize that global health security begins locally – if there are gaps anywhere in the system, disease will find it.

Lessons learned through the lens of the GDD program can offer us a way forward. More than a decade of successes and failures has given us information and evidence-based strategies essential to developing core public health capacities around the world. These strategies include increasing coordinated, multi-center scientific collaboration across nations to strengthen the global network; increasing the number of public health professionals trained; broadening and strengthening global partnerships; and reducing gaps in global preparedness for emerging health threats.

As the global health community looks for the best ways to operate in our changing world, lessons from the GDD program will continue to inform our work. We have an obligation to keep our nation and our world safe, healthy, and secure. We must therefore continue our efforts — and commit to doing much more — to improve what we can, where we can, on a continual basis. We can afford nothing less.
